# Iron-Catalyzed Oxidation of 1-Phenylethanol and Glycerol With Hydrogen Peroxide in Water Medium: Effect of the Nitrogen Ligand on Catalytic Activity and Selectivity

**DOI:** 10.3389/fchem.2020.00810

**Published:** 2020-10-09

**Authors:** Dimitri Ros, Teresa Gianferrara, Corrado Crotti, Erica Farnetti

**Affiliations:** ^1^Dipartimento di Scienze Chimiche e Farmaceutiche, Università di Trieste, Trieste, Italy; ^2^Unità Operativa di Supporto di Trieste, Istituto Struttura della Materia, Consiglio Nazionale delle Ricerche, Trieste, Italy

**Keywords:** iron catalysts, nitrogen ligands, oxidation, alcohols, glycerol

## Abstract

The iron(II) complexes [Fe(bpy)_3_](OTf)_2_ (bpy = 2,2'-bipyridine; OTf = CF_3_SO_3_) (**1**) and [Fe(bpydeg)_3_](OTf)_2_ (bpydeg = *N*^4^,*N*^4^-bis(2-(2-methoxyethoxy)ethyl) [2,2'-bipyridine]-4,4'-dicarboxamide) (**2**), the latter being a newly synthesized ligand, were employed as catalyst precursors for the oxidation of 1-phenylethanol with hydrogen peroxide in water, using either microwave or conventional heating. With the same oxidant and medium the oxidation of glycerol was also explored in the presence of **1** and **2**, as well as of two similar iron(II) complexes bearing tridentate ligands, *i.e*., [Fe(terpy)_2_](OTf)_2_ (terpy = 2, 6-di(2-pyridyl)pyridine**)** (**3**) and [Fe(bpa)_2_](OTf)_2_ (bpa = bis(2-pyridinylmethyl)amine) (**4**): in most reactions the major product formed was formic acid, although with careful tuning of the experimental conditions significant amounts of dihydroxyacetone were obtained. Addition of heterocyclic amino acids (*e.g.*, picolinic acid) increased the reaction yields of most catalytic reactions. The effect of such additives on the evolution of the catalyst precursors was studied by spectroscopic (NMR, UV-visible) and ESI-MS techniques.

## Introduction

The ability of iron to promote oxidation of a variety of organic molecules has been known since the 19th century, but only in the last two decades has it been the object of intense research. Thus, an impressive number of papers has appeared in the literature, concerning the design and properties of various iron catalysts, most of which bear nitrogen chelating ligands, mimicking the active sites of redox iron-based enzymes (Engelmann et al., 2016; Oszajca et al., 2016; Sahu and Goldberg, 2016). Although, also a moderate number of ligand-bearing donor atoms other than nitrogen have been successfully employed in association to iron for oxidation reactions (Lenze and Bauer, 2009; Rani and Bhat, 2010; Farnetti et al., 2020), however such complexes generally show inferior catalytic properties in comparison to bioinspired iron derivatives with nitrogen ligands. In this view, much effort has been spent in the synthesis of novel nitrogen ligands which might tune the iron oxidation properties.

Alcohol oxidation to the corresponding carbonylic compounds is an important chemical process in which iron catalysis has been playing a leading role. In contrast to traditional oxidation methods, the use of iron derivatives is a key feature for the development of sustainable catalytic reactions, due to large availability, low price, and negligible toxicity of this metal (Lenze et al., 2013a,b; Szávuly et al., 2014; Tan et al., 2015; Martins et al., 2016; Neve et al., 2016; Olivo et al., 2016; Pinto et al., 2016; Sheet and Paine, 2016; Bauer, 2017; Yan et al., 2017). Of course, in order to assess a thoroughly green process other reaction features must be accordingly chosen, among which the nature of the oxidant and of the reaction medium are especially relevant.

Recent research in catalytic oxidation has been almost exclusively concerned with the use of either molecular oxygen or peroxides as oxidizing agents. Molecular oxygen, employed by enzymes in biologic processes, certainly represents the greenest choice; however, use of O_2_ in laboratory reactions often proves to be difficult to control, not seldom resulting in highly selective reactions yielding overoxidized products. On the other hand, peroxides such as H_2_O_2_ and *tert*-butylhydroperoxide (TBHP) are moderately priced, have low toxicity oxidants, and the nature of their byproducts (water and alcohol, respectively), makes them highly sustainable reagents (Talsi and Bryalov, 2012). Nevertheless, when using peroxides in the presence of an iron catalyst one must be aware that such metal promotes peroxide degradation, thus partly consuming the oxidizing agent (Menage et al., 1994).

With regard to the reaction medium, in the last few years the progressive growth of awareness of environmental issues has stimulated remarkable efforts toward the use of green solvents (Sheldon, 2012) or, preferably, of either an aqueous medium or solvent-less conditions.

Solvent selection appears to be especially tricky when the oxidation of glycerol is considered. Valorization of this molecule, which is largely available being the byproduct of biodiesel synthesis, is of considerable commercial interest (Pagliaro et al., 2007; Behr et al., 2008; Zhou et al., 2008; Diaz-Alvarez et al., 2011; Tran and Kamali Kannangara, 2013). Unfortunately, on one hand the physical properties of this polyalcohol (high viscosity, low solubility in most organic solvents) makes it difficult to select suitable experimental conditions, but on the other its very polyfunctionality represents a challenge toward the development of selective reactions. With regard to the selection of the solvent when using glycerol as substrate, water would be by far the best choice, from the point of view of both solubility and sustainability. Moreover, the association of a peroxide as an oxidizing agent with an aqueous medium looks highly appropriate.

From the observations above reported, the development of iron-based catalysts bearing water soluble ligands appears highly desirable. Our group has been recently involved in investigations concerning sustainable iron-catalyzed oxidation of alcohols using peroxides as oxidizing agents; in this field, part of the studies dealt with the selective oxidation of glycerol. So far, most of our studies employed iron derivatives with commercial bi- or tri-dentate nitrogen ligands: among those examined, complexes of the type [Fe(bpy)_3_](OTf)_2_ (bpy = 2,2'-bipyridine; OTf = CF_3_SO_3_) (**1**) and its analogs with substituted nitrogen-chelating ligands proved to be effective catalysts for the oxidation of alcohols with peroxides, either in organic solvent (acetonitrile, acetone) or in mixtures solvent/water (Chavez et al., 2016; Cozzi et al., 2018). Use of this class of catalysts in water with no addition of organic solvent would be desirable, both from the point of view of sustainability as well as in view of a possible application to the oxidation of glycerol; unfortunately, water solubility of **1** and similar species proved to be either limited or poor. We were attracted by the possibility of increasing the solubility in water of this class of complexes by decorating the nitrogen ligands with highly hydrophilic groups. For this purpose, the bpy derivative N^4^,N^4^-bis(2-(2-methoxyethoxy)ethyl)[2,2'-bipyridine]-4,4'-dicarboxamide (bpydeg) was synthesized and its corresponding iron derivative was obtained by a reaction with iron(II) triflate.

In the following, we describe the synthesis of the novel ligand bpydeg as well as of its iron complex [Fe(bpydeg)_3_](OTf)_2_ (**2**); both bpy and bpydeg derivatives were employed as catalyst precursors for the oxidation of 1-phenylethanol and glycerol, using hydrogen peroxide in a water medium. In order to extend the investigation on the effect of the nature of nitrogen ligand on the activity and selectivity in glycerol oxidation, the complexes [Fe(terpy)_2_](OTf)_2_ (terpy = 2,6-di(2-pyridyl)pyridine) (**3**) and [Fe(bpa)_2_](OTf)_2_ (bpa = bis(2-pyridinylmethyl)amine) (**4**) were in turn employed as catalyst precursors. A comparison of the catalytic properties of the four iron complexes is reported, together with the results of spectroscopic studies concerning the evolution of the bpa derivative **4** under experimental conditions similar to those employed in the catalytic reactions.

## Materials and Methods

### General

All the chemicals were reagent grade and were used as received from the commercial suppliers. The iron complexes **1** and **4** were synthesized according to published procedures (Lenze et al., 2013a; Chavez et al., 2016).

### Instrumental

^1^H and ^13^C NMR spectra were recorded either on a Varian 500 spectrometer or on a Varian 400 spectrometer; ^19^F NMR spectra were recorded on a Varian 400 spectrometer. Chemical shifts were measured relative to the residual solvent signal. Resonances were assigned with reference to COSY and HSQC spectra.

UV-visible spectra were recorded on a Shimadzu UV-2450 spectrophotometer equipped with a TCC-240A temperature-controlled cell holder.

ESI-MS spectra were obtained by an ion-trap instrument (ESI-MS Bruker Esquire 4000) equipped with an electrospray ion source. The instrument performed with 10.0 psi nebulizer pressure, end-plate offset −500 V, capillary 4,000 V, and capillary exit at 113.3 V. The drying gas (N_2_) flow was 5 L min^−1^ and the spectral range was from *m/z* = 100–1,500.

The catalytic reactions were performed either in a thermostatted bath or using a CEM Discover Labmate microwave reactor.

The chemical yields of the catalytic reactions were determined by the integration of the ^1^H NMR signals and/or by GC analysis on an Agilent 6850 instrument with helium as the carrier gas and a TCD detector. Samples from the reaction mixtures were injected without previous dilution at 100°C into the cool on-column injector (“track-oven” programmed temperature) in a Restek Rtx®-Wax capillary column (30 m length, 0.32 mm ID, 0.5 μm film thickness) protected by a Restek Hydroguard® FS precolumn (5 m length, 0.53 mm ID).

### Synthesis of *N*^4^,*N*^4^-bis(2-(2-methoxyethoxy)ethyl)[2,2'-bipyridine]-4,4'-dicarboxamide (bpydeg)

#### Synthesis of 2-(2-methoxyethoxy)ethyl Tosylate

In a round bottom flask, 7.2 g of tosyl chloride (TsCl) (38 mmol) were dissolved in 10 mL of anhydrous dichloromethane. Upon vigorous stirring at 0°C, 4.60 mL of diethylene glycol monomethyl ether (38 mmol) and 10 mL of triethylamine (TEA) (72 mmol) dissolved in 10 mL of anhydrous dichloromethane were added over 20 min under argon. The mixture was allowed to warm at r. t. and it was left under stirring for 18 h. A white water soluble precipitate was formed. The suspension was extracted with water (1 × 15 mL) and the aqueous phase was washed with dichloromethane (2 × 15 mL). The organic fractions were combined and washed with 6M HCl (2 × 10 mL), 5% NaHCO_3_ (2 × 10 mL), and water (2 × 10 mL). After drying over Na_2_SO_4_, the organic layer was evaporated under reduced pressure to give 8.54 g of a pale yellow oil. Yield 82%. ^1^H NMR (400 MHz, CDCl_3_), δ 7.79 (d, 2H, S-C=*CH*-CH), 7.33 (d, 2H, S-C=CH-*CH*), 4.16 (t, 2H, OCH_2_*CH*_2_OTs), 3.68 (t, 2H, O*CH*_2_CH_2_OTs), 3.57 (m, 2H, OCH_2_CH_2_O), 3.47 (m, 2H, OCH_2_CH_2_O), 3.34 (s, 3H, CH_3_), 2.44 (s, 3H, C-CH_3_).

#### Synthesis of 2-(2-methoxyethoxy)ethyl Azide

To 4.33 g (16 mmol) of 2-(2-methoxyethoxy)ethyl tosylate in 50 mL of DMSO, 2.08 g (32 mmol) of sodium azide (NaN_3_) were added and the resulting suspension was stirred under argon for 18 h. The resulting solution was added with 5 mL of water and left under stirring at r. t. for a further 30 min. The solution was extracted with ethyl ether (3 × 50 mL), the organic fractions were combined and evaporated to dryness to give 1.84 g of a colorless oil. Yield 80%. ^1^H NMR (400 MHz, CDCl_3_), δ 3.67 (m, 4H, CH_2_OCH_2_), 3.56 (m, 2H, CH_3_OC*H*_2_), 3.41 (t, 2H, CH_2_N_3_), 3.38 (s, 3H, CH_3_).

#### Synthesis of 2-(2-methoxyethoxy)ethylamine

3.60 g (14 mmol) of triphenylphosphine (TPP) were added to 1.81 g (13 mmol) of 2-(2- methoxyethoxy)ethyl azide dissolved in 25 ml of ethyl ether and cooled to 0°C. The suspension was left under stirring under argon for 1 h at 0°C and for 1.5 h at r. t. The reaction was quenched with 5 ml of water and the mixture was stirred for 18 h at r. t. The white precipitate of triphenylphosphinoxide was removed by washing the reaction mixture with toluene (3 × 25 ml). The aqueous phase was concentrated under vacuum to a minimum volume and 50 ml of dichloromethane and anhydrous Na_2_SO_4_ were added to the residue obtained. after 12 h, the solid was removed by filtration and the solution was evaporated under reduced pressure, obtaining 0.89 g of the free amine as yellowish oil. Yield 60%. ^1^H NMR (400 MHz, CDCl_3_), δ 3.54 (m, 6H, (CH_2_)_2_O and *CH*_2_–CH_2_-NH_2_), 3.37 (s, 3H, CH_3_), 2.86 (t, 2H, *CH*_2_NH_2_), 1.34 (s, 2H, NH_2_).

#### Synthesis of N^4^,N^4^-bis(2-(2-methoxyethoxy)ethyl)[2,2'-bipyridine]-4,4'-dicarboxamide

Five hundred mg (2.05 mmol) of 2,2'-bipyridyl 4,4'-dicarboxylic acid, 1.78 g (9.28 mmol) of *N*-ethyl-*N*′-(3-dimethylaminopropyl)carbodiimide hydrochloride (edci), and 830 mg (6.14 mmol) of 1-hydroxybenzotriazole (HOBt) were dissolved in 15 mL of DMF. The resulting solution was kept under stirring at room temperature for 30 min. Then 537 mg (4.5 mmol) of 2-(2-methoxyethoxy)ethylamine and 550 mg (4.5 mmol) of 4-(dimethylamino)pyridine (DMAP) in 1 ml of DMF were added and the reaction was left under stirring for 48 h at r.t. To monitor the reaction, 50 μl were taken at timed intervals, the DMF was evaporated under reduced pressure, and the residue dissolved in dichloromethane was applied on a silica gel TLC plate and eluted with dichloromethane/ethanol 7:3. At the end of the reaction, the DMF was evaporated under vacuum, the residue was dissolved in 50 ml of chloroform and washed with water (1 × 25 mL). The organic phase was concentrated and applied on a silica gel column and eluted with dichloromethane/ethanol 9:1, then with 8.2 to obtain 707 mg of a white powder. Yield 77%. ^1^H NMR (400 MHz, CDCl_3_), δ 8.81 (d, 2H, ${\text{H}}_{6,6^{\text{′}}}$ bpy), 8.72 (s, 2H, ${\text{H}}_{3,3^{\text{′}}}$ bpy), 7.79 (dd, 2H, ${\text{H}}_{5,5^{\text{′}}}$ bpy), 7.08 (br m, 2H, CONH), 3.72 (m, 8H, CH_2_O), 3.69 (m, 4H, *CH*_2_CH_2_NHCO), 3.58 (m, 4H, *CH*_2_NHCO), 3.38 (s, 6H, CH_3_).

### Synthesis of Iron Complexes 2 and 3

#### Synthesis of [Fe(bpydeg)_3_](OTf)_2_ (2)

A round-bottomed flask was charged with acetonitrile (3 mL) and Fe(OTf)_2_ (80 mg, 0.22 mmol), the pale yellow solution so obtained turned deep violet upon the addition of bpydeg (200 mg, 0.45 mmol). The reaction mixture was stirred at r.t. for 1 h and then concentrated to half volume. The addition of diethyl ether caused the precipitation of a violet solid, which was filtered and washed with ether. Yield 77%. ^1^H NMR (CD_3_CN, 25°C), δ 8.96 (d, 6H, ${\text{H}}_{3,3^{\text{′}}}$), 7.70 (dd, 6H, ${\text{H}}_{6,6^{\text{′}}}$), 7.66 (brs, 6H, NH), 7.55 (m, 6H, ${\text{H}}_{5,5^{\text{′}}}$), 3.65, 3.60, and 3.27 (m, 48H, CH_2_), 3.27 (s, 18H, Me). ^19^F NMR (CD_3_CN, 25°C) δ -79.6.

#### Synthesis [Fe(terpy)_2_](OTf)_2_ (3)

In a round-bottomed flask acetonitrile (15 mL) was added to Fe(OTf)_2_ (200 mg, 0.56 mmol), the resulting solution turned deep violet upon the addition of terpy (264 mg, 1.13 mmol). The reaction mixture was stirred at r.t. for 1 h and then concentrated to half volume. The addition of diethyl ether caused a precipitation of a violet solid, which was filtered and washed with ether. Yield 70%. ^1^H NMR (CD_3_CN, 25°C), δ 8.92 (d, 2H, H_3_ +H_5_), 8.69 (t, 1H, H_4_), 8.48 (d, 2H, ${\text{H}}_{3,3^{\text{′}}}$), 7.89 (dt, 2H, ${\text{H}}_{4^{\text{′}},4^{\prime \prime }}$), 7.08 (d, 4H, ${\text{H}}_{5^{\text{′}},5^{\prime \prime }}$ + ${\text{H}}_{6^{\text{′}},6^{\prime \prime }}$). ^19^F NMR (CD_3_CN, 25°C) δ -79.4.

### Catalytic Reactions

#### Oxidation of 1-phenylethanol or Glycerol Using Microwave Heating

A MW vial was charged with the solvent (0.65 mL), the catalyst (7.5 × 10^−3^ mmol), the substrate (0.75 mmol), and finally the oxidant. The vial was then immediately placed into the microwave reactor and heated to the fixed temperature under magnetic stirring. After the desired time the reaction mixture was cooled at r.t. and subsequently analyzed by GC and/or ^1^H NMR.

#### Oxidation of Glycerol Using Conventional Heating

In a round-bottomed flask the solvent (0.65 mL) and the catalyst (0.010 mmol) were introduced, followed by the substrate (0.50 mmol). For reactions performed at temperatures higher than r.t., the resulting solution was heated in a thermostatted bath to the desired temperature. Slow addition of the oxidant was then carried out under stirring. After the desired time, the reaction mixture was cooled at r.t. and analyzed by GC and/or ^1^H NMR.

### Analysis of the Reaction Mixtures

Qualitative analysis of the crude reaction mixtures was accomplished by ^1^H and ^13^C NMR and GC; the NMR resonances and the GC retention times were compared to those of authentic samples obtained either by conventional routes or by commercial suppliers. Formation of other possible oxidation products (*e.g.*, carboxylic acids) was ruled out due to the absence of both NMR signals and GC peaks of known retention times, obtained from authentic samples. Quantitative analysis was also performed on the crude reaction mixtures by the integration of the ^1^H NMR signals and/or by GC using response factors previously determined by the analysis of standard solutions (internal standard: *tert*-butanol); the quantitative analysis thus performed allowed for a reproducibility within ±1%

### Scale-up of Glycerol Oxidation Catalyzed by (4)

In a reaction flask 0.040 mmol of **4** were dissolved in 1.4 mL D_2_O and then treated with 1 mL of a 1.7 M glycerol solution in D_2_O. The slow addition of 2 eq of H_2_O_2_ 30% was accomplished in two portions at time intervals of 15 min. After a further 15 min (total time 30 min after initial addition) the reaction mixture was treated with 500 mg of Na_2_SO_3_ then the solvent was evaporated under reduced pressure. The residue was dissolved in acetonitrile and purified by chromatography on silica gel using acetonitrile as the mobile phase. The appropriate fractions were evaporated to dryness, yielding a colorless liquid (theoretical yield 27.6 mg, isolated yield 22.6 mg).

## Results and Discussion

### Synthesis of bpydeg and [Fe(bpydeg)_3_](OTf)_2_ (2)

As mentioned in the introduction section, in previous studies (Chavez et al., 2016; Cozzi et al., 2018) we observed that [Fe(bpy)_3_](OTf)_2_ (**1**) behaved as an active catalyst for the oxidation of secondary alcohols to the corresponding ketones by use of peroxides. The catalytic reactions could be performed either in an organic solvent (acetonitrile, acetone), in mixtures solvent/water, or in an aqueous medium, the latter of course being the preferred choice from the point of view of sustainability. Thus, we reasoned that the decoration of bpy with hydrophilic groups might provide a ligand, and its corresponding iron complex, which was expected to be more soluble in water, and possibly more effective for catalytic oxidation in such a medium. In this perspective, the ligand N^4^,N^4^-bis(2-(2-methoxyethoxy)ethyl)[2,2'-bipyridine]-4,4'-dicarboxamide (bpydeg, Scheme 1) was chosen as an appropriate candidate, and synthesized according to the procedure described in the following.

**Figure 1:**
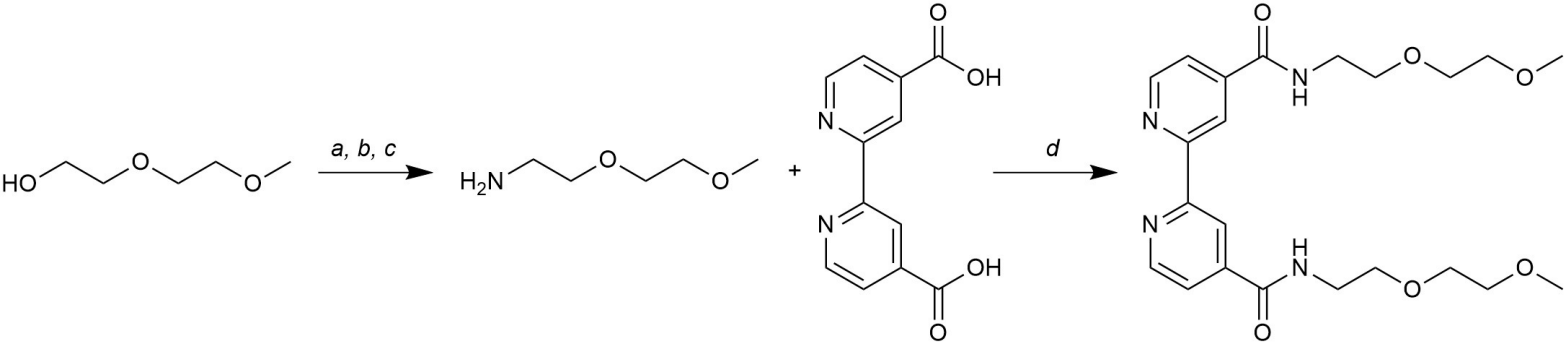
Synthetic route to bpydeg. Reactions and conditions: **(a)** TsCl, TEA, 0°C, 20 min, r. t., 18 h (82%); **(b)** NaN_3_, DMSO, r. t., 18 h (80%); **(c)** TPP, Et_2_O, 0°C, 1 h, r.t., 1.5 h; H_2_O r. t., 18 h (60%); **(d)** EDCI/HOBt, DMF, r. t., 30 h (77%).

The synthetic route for obtaining this new ligand is shown in Figure 1. First, 2-(2-methoxyethoxy)ethylamine was synthesized with a multistep procedure. Diethylene glycol monomethyl ether was tosylated and then a nucleophilic displacement with sodium azide gave 2-(2- methoxyethoxy)ethyl azide. A biphasic Staudinger reaction, a very mild azide reduction (Neumayer et al., 1998), gave the corresponding amine compound. The 2-(2-methoxyethoxy)ethylamine was reacted with the hydroxybenzotriazole (HOBt) ester of 2,2'-bipyridine-4 4'-dicarboxylic acid in coupling conditions (Montalbetti and Falque, 2005) to give bpydeg in good yield.

**Figure 2:**
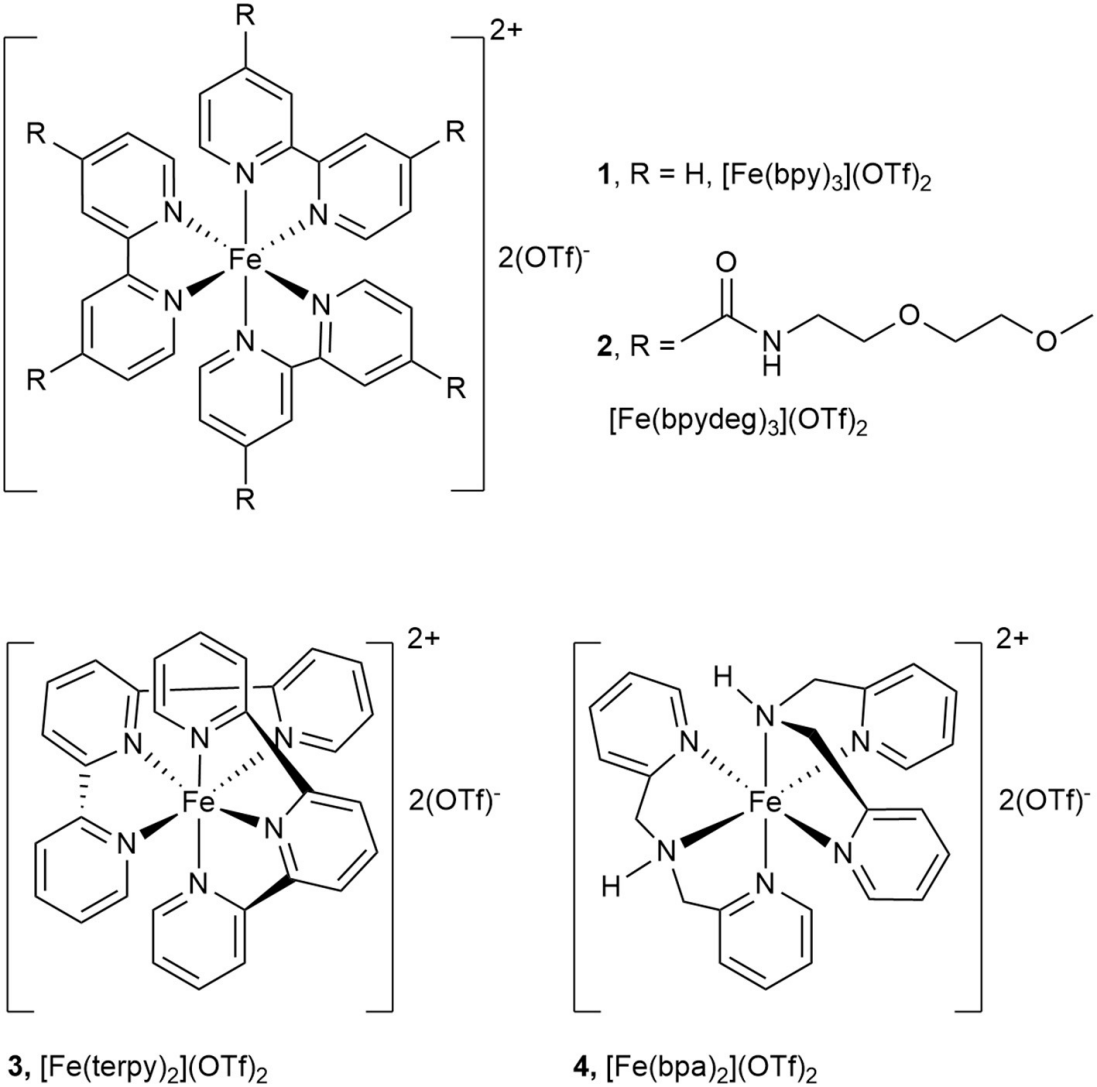
Complexes [Fe(bpy)_3_](OTf)_2_ (**1**), [Fe(bpydeg)_3_](OTf)_2_ (**2**), [Fe(terpy)_2_](OTf)_2_ (**3**), and [Fe(bpa)_2_](OTf)_2_ (**4**).

For the synthesis of [Fe(bpydeg)_3_](OTf)_2_ (**2**) the same procedure previously employed to prepare [Fe(bpy)_3_](OTf)_2_ (**1**) (Chavez et al., 2016) was followed: to a solution of Fe(OTf)_2_ in acetonitrile 2 eq of the ligand were added, causing the immediate formation of a deeply violet colored solution. After 30 min at r.t. the solution was concentrated, then the addition of ether caused the precipitation of the desired compound **2** as a violet solid (see Figure 2). The ^1^H NMR spectrum of a CD_3_CN solution of **2** showed in the aromatic region a doublet at δ 8.96 and a double doublet at δ 7.70, assigned to ${\text{H}}_{3,3^{\text{′}}}$ and ${\text{H}}_{6,6^{\text{′}}}$, respectively, aside a broad signal at δ 7.55 (NH) and a multiplet at δ 7.55 assigned to ${\text{H}}_{5,5^{\text{′}}}$. At higher field, besides multiplets at δ 3.65, 3.60, and 3.27 due to polyglycol chain protons, a singlet at δ 3.27 was assigned to methyl protons. In the ^19^F NMR spectrum a narrow signal at δ−79.6 indicated the presence of non-coordinated triflate ion.

### Oxidation of 1-phenylethanol With H_2_O_2_ Catalyzed by [Fe(L)_3_](OTf)_2_ (L=bpy, bpydeg) With MW Heating

We tested the catalytic properties of the newly synthesized complex **2** in promoting the oxidation of 1-phenylethanol to acetophenone. The reactions were performed using hydrogen peroxide as the oxidizing agent and MW heating which had been successfully employed in previous studies (Cozzi et al., 2018). The most significant results of catalytic reactions performed at 100°C with complex 2 and, for comparison, with the bpy derivative **1** are reported in Table 1. Use of acetonitrile as the solvent resulted in moderate conversions with both catalysts, with **2** being somewhat superior to **1** (70 vs. 62%, see entries 1 and 2 in Table 1). With the addition of an acidic buffer to the organic solvent an increase of reaction yields to 81% was obtained (entry 3 in Table 1): use of a buffer at pH=1 was previously explored, and its positive effect on the catalytic reaction was ascribed to the lower degree of iron promoted degradation of hydrogen peroxide usually observed in acidic solutions, in comparison to neutral or basic conditions (Cozzi et al., 2018). The catalytic reaction carried out with **2** using only the buffer gave a similar yield (82%) to what was obtained in the buffer-acetonitrile mixture, to be compared with 67% obtained with catalyst **1** (see Table 1 entries 5 and 4, respectively). When the same reaction with complex **2** was repeated in pure water the conversion was lower (Table 1, entry 6), once more proving the beneficial effect of an acidic medium. On the other hand, the use of a double-fold amount of oxidant caused only a negligible increase in the reaction yield (in Table 1 compare entries 7 and 5).

**Table 1:** Oxidation of 1-phenylethanol with H_2_O_2_ catalyzed by [Fe(L)_3_](OTf)_2_ with MW heating^a^.

**Entry**	**L**	**Solvent**	**[H_**2**_O_**2**_]/[sub]**	**Conv. (%)**
1	bpy	CH_3_CN	4	62
2	bpydeg	CH_3_CN	4	70
3	bpydeg	CH_3_CN/buffer^b^^,^^c^	4	81
4	bpy	buffer^b^	4	67
5	bpydeg	buffer^b^	4	82
6	bpydeg	water	4	71
7	bpydeg	buffer^b^	8	83
8^d^	bpydeg	buffer^b^	4	81

a *Experimental conditions: [Fe]= 1.2 × 10^−2^ M; [sub]/[Fe]= 100; H_2_O_2_(aq) 30%; T = 100°C; t = 30 min*.

b Buffer HCl/KCl (pH = 1.0);

c *CH_3_CN: buffer = 1:4*.

d *cocat = Hpic; [Hpic]/[Fe] = 5*.

**Figure 3:**
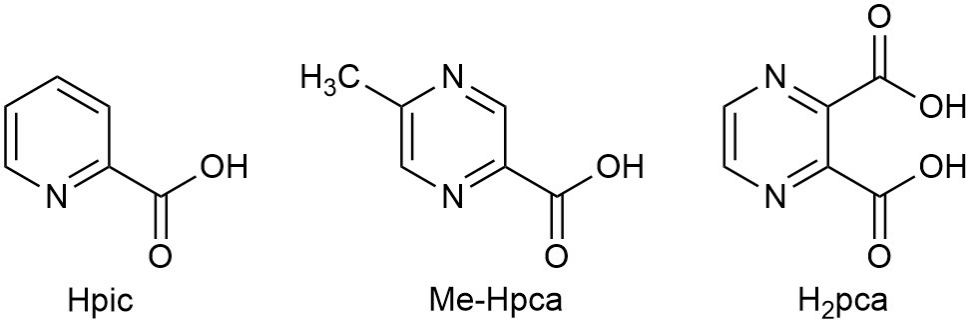
Additives.

We then explored the effect on the catalytic reaction of an addition of a cocatalyst, chosen among a series of heterocyclic amino acids which are known to be good candidates in enhancing the catalytic properties of iron catalysts according to several authors' studies as well as ours (Shul'pin, 2002, 2013; Fernandes et al., 2011; Join et al., 2011; Kirillov and Shul'pin, 2013; Tanaka et al., 2014; Cozzi et al., 2018). Thus, it was ascertained that in the reactions under investigation such compounds did not appear to play a significant role as cocatalysts, as exemplified by the single entry 8 in Table 1 (81%), referring to the use of 2-pyridinecarboxylic acid (Hpic), to be compared to entry 5 (82%). Other additives such as 2-pyrazinecarboxylic acid (H_2_pca) and 5-methyl-2-pyrazinecarboxylic acid (Me-Hpca) (see Figure 3) gave similar results.

All the data reported in Table 1 are referred to a reaction time of 30 min, as we ascertained that longer times failed to provide increased reaction yields. Also the chosen reaction temperature (100°C) proved to be the appropriate choice in terms of final conversion, as higher temperatures did not produce significantly higher reaction yields.

### Oxidation of Glycerol With H_2_O_2_ Catalyzed by [Fe(L)_3_](OTf)_2_ (L=bpy, bpydeg) With MW Heating

The iron derivative **2**, which proved to be effective in promoting oxidation of the model substrate 1-phenylethanol in a water medium, was then employed for the oxidation of a more interesting although challenging substrate, *i.e.*, glycerol. Such molecules, as mentioned in the introduction section, can be oxidized to a variety of products, all of which are of value from an industrial point of view. However, glycerol oxidation raises a main difficulty concerning the reaction selectivity, as in most cases a mixture of oxidation products is obtained. Moreover, the most valuable product, dihydroxyacetone (DHA), is susceptible to degradation when exposed to relatively high pH or temperature (Crotti et al., 2010).

**Figure 4:**
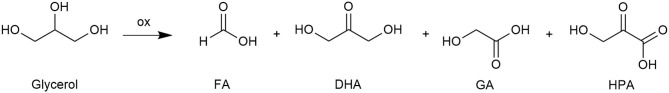
Products of glycerol oxidation.

In the present study, the catalytic oxidations of glycerol with MW heating were carried out in water using the buffer at pH=1 previously employed, which gave better results than non-buffered water in all experimental conditions; once more, hydrogen peroxide was used as the oxidant. All reactions yielded a mixture of four products (see Figure 4): minor amounts of DHA, glycolic acid (GA), hydroxypyruvic acid (HPA), aside the major product formic acid (FA).

The most significant results obtained with catalysts **1** and **2** are reported in Table 2. The reaction temperature had no significant effect on the overall conversion and a negligible effect on selectivity (see Table 2 entries 1-3); in all experimental conditions complex **1** was somewhat superior to **2** (in Table 2 compare entries 3 and 4, 5 and 6, 7, and 8). Higher amounts of hydrogen peroxide caused an increase of glycerol oxidation but a loss in terms of selectivity as formation of formic acid was favored (see reactions 3, 5, 7 and 4, 6, 8 in Table 2). Finally, conversions close to 100% could be obtained with higher amounts of oxidant and longer reaction times, however in such cases the only product formed was formic acid.

**Table 2:** Oxidation of glycerol with H_2_O_2_ catalyzed by [Fe(L)_3_](OTf)_2_ with MW heating^a^.

**Entry**	**L**	**T (^**°**^C)**	**[H_**2**_O_**2**_]/[sub]**	**Conv. (%)**	**Yields FA (%)**	**GA (%)**	**HPA (%)**	**DHA (%)**
1	bpydeg	60	1	40	29	1	2	8
2	bpydeg	80	1	41	30	2	2	7
3	bpydeg	100	1	43	31	3	3	6
4	bpy	100	1	56	38	5	6	7
5	bpydeg	100	1.5	59	46	3	4	6
6	bpy	100	1.5	64	48	5	5	6
7	bpydeg	100	2	75	61	4	5	5
8	bpy	100	2	78	60	6	6	6

a *Experimental conditions: [Fe]= 1.2 × 10^−2^ M; [sub]/[Fe]= 100; H_2_O_2_(aq) 30%; t = 30 min; solvent = buffer HCl/KCl (pH = 1.0). FA, formic acid; DHA, dihydroxyacetone; GA, glycolic acid; HPA, hydroxypyruvic acid*.

### Oxidation of Glycerol With H_2_O_2_ Catalyzed by [Fe(L)_3_](OTf)_2_ (L=bpy, bpydeg) With Conventional Heating

As discussed in Section Oxidation of Glycerol with H_2_O_2_ Catalyzed by [Fe(L)3](OTf)2 (L=bpy, bpydeg) With MW Heating, complexes **1** and **2** catalyzed the oxidation of glycerol with hydrogen peroxide and microwave heating with good catalytic activity although low selectivity, as the main product formed was formic acid in all cases. More in detail, the desired product DHA was formed in low yields, and its amount decreased at higher temperatures and longer reaction times: such results are coherent with the known thermal degradation of DHA, as well as its susceptibility to be further oxidized. We then reasoned that, possibly, the use of conventional heating and lower reaction temperatures would be more favorable in order to enhance the formation of DHA.

Therefore, oxidation of glycerol was carried out in the presence of catalysts **1** and **2**, using hydrogen peroxide and heating by means of a thermostatted oil bath. After the first set of reactions it was apparent that in these experimental conditions the use of an acidic buffer as a reaction medium gave similar results to non-buffered water, therefore the data reported in Table 3 are referred to catalytic reactions performed in pure water. Some tests performed at r.t. yielded only traces of DHA as the only product (see Table 3, entries 1 and 2); reactions performed at 40°C gave similar results with 1 eq of H_2_O_2_ (see entry 3 in Table 3), whereas, a double-fold amount of oxidant produced an increase of conversion, however the main product formed was formic acid (Table 3, entries 4 and 5). An increase of temperature to 60°C produced higher yields of both DHA and FA, but the latter product was the most abundant one in all cases, limiting the final yield of DHA to 7% or less (see entries 6-8 in Table 3); moreover, at such a temperature or higher, moderate amounts of glycolic acid were also formed, together with traces of hydroxypyruvic acid. Reactions 4, 5, 7, and 8 were also carried out with a modification of experimental procedure, i.e., hydrogen peroxide was added in two portions at a time interval of 15 min, an expedient that, according to our previous studies, can prove effective in order to reduce the extent of iron-promoted peroxide decomposition (catalase-like activity): unfortunately, no difference in both conversions and selectivities were observed.

**Table 3:** Oxidation of glycerol with H_2_O_2_ catalyzed by [Fe(L)_3_](OTf)_2_ with conventional heating^a^.

**Entry**	**L**	**T (^**°**^C)**	**[H_**2**_O_**2**_]/[sub]**	**Conv. (%)**	**Yields FA (%)**	**GA (%)**	**DHA (%)**
1	bpy	25	2	2	–	–	2
2	bpydeg	25	2	2	–	–	2
3	bpy	40	1	3	–	–	3
4	bpy	40	2	19^b^	16	<1	3
5	bpydeg	40	2	18	17	–	<1
6	bpy	60	1	22^b^	18	<1	4
7	bpy	60	2	44^b^	33	4	7
8	bpydeg	60	2	54^b^	43	5	6
9^c^	bpy	60	2	56^b^	45	4	7
10	bpy	80	2	48^b^	37	3	7

a *Experimental conditions: [Fe]= 1.5 × 10^−2^ M; [sub]/[Fe] = 50; H_2_O_2_(aq) 30%; t = 30 min; solvent = water. FA, formic acid; DHA, dihydroxyacetone; GA, glycolic acid*.

b *Also formed hydroxypyruvic acid <1%*.

c *cocat = Hpic; [Hpic]/[Fe] = 5*.

Also the use of the additive Hpic was not effective, as it caused an increase of only formic acid yield (compare entries 7 and 9 in Table 3). On the other hand, the addition of free ligand, which in previous work was found to cause an increase in DHA selectivity (Crotti and Farnetti, 2014) was not favorable in this series of reactions, as it caused a decrease of overall conversion but no increase of DHA yield: for example, using catalyst **1** in the presence of 3 eq of bpy at 60°C final yields were 20% (FA) and 5% (DHA), to be compared to 33 and 7%, respectively (entry 7 in Table 3).

A further increase of reaction temperature to 80°C produced no significant changes (Table 3, entry 10). In all reactions performed, similar results were obtained with the two catalysts **1** and **2**, indicating a negligible effect of bpy decoration on the catalyst performance.

### Oxidation of Glycerol With H_2_O_2_ Catalyzed by [Fe(L_2_](OTf)_2_ (L=terpy, bpa) With Conventional Heating

The results described in Oxidation of Glycerol With H2O2 Catalyzed by [Fe(L)3](OTf)2 (L=bpy, bpydeg) With Conventional Heating indicate that the ligands bpy and bpydeg in association with iron form active catalysts for glycerol oxidation, although their selectivity was disappointing as the main product observed was formic acid. In order to further investigate the effect of the nature of nitrogen ligand on catalytic activity and selectivity, these studies were extended to the use of the two tridentate ligands terpy and bpa (see Figure 2).

Thus, the complex [Fe(terpy)_2_](OTf)_2_ (**3**) (see Figure 2) was synthesized from Fe(OTf)_2_ and 2 eq of the ligand, in acetonitrile: the addition of diethyl ether to the concentrated solution afforded a dark purple solid. The ^1^H NMR spectrum of its CD_3_CN solution showed two signals assignable to the protons of the central ring (δ 8.92 and 8.69) together with other signals due to terminal rings (δ 8.48, 7.97, 7.89, and 7.08). In the ^19^F NMR spectrum, a signal at δ−79.4 indicated that triflate ion is neither coordinated to iron nor involved in an equilibrium with the complex.

On the other hand, the complex [Fe(bpa)_2_](OTf)_2_ (**4**) was prepared according to the published procedure (Lenze et al., 2013a). Such iron species had been employed in our previous studies concerning glycerol oxidation in organic solvent, giving promising results in terms of selectivity in DHA formation (Crotti and Farnetti, 2014).

Complexes **3** and **4** were employed as catalysts for oxidation of glycerol with hydrogen peroxide in water. Conventional heating was used when appropriate. A selection of the results obtained is reported in Table 4.

**Table 4:** Oxidation of glycerol with H_2_O_2_ catalyzed by [Fe(L)_2_](OTf)_2_ with conventional heating^a^.

**Entry**	**L**	**Cocat^**b**^**	**T (^**°**^C)**	**T (min)**	**[H_**2**_O_**2**_]/[sub]**	**Conv. (%)**	**Yields FA (%)**	**GA (%)**	**DHA (%)**
1	terpy	–	40	30	2	30	25	2	3
2	terpy	–	60	30	1	26	21	<1	4
3	terpy	–	60	30	2	43	33	2	8
4	terpy	Hpic	60	30	2	51	40	2	9
5	bpa	–	25	15	2	33	23	<1	10
6^c^	bpa	–	25	15	2	16	5	-	11
7^c^	bpa		25	15	3	25	11	-	14
8	bpa	Hpic	25	15	2	47	31	<1	16
9	bpa	H_2_pca	25	15	2	54	35	7	12
10	bpa	Me-Hpca	25	15	2	46	30	4	12
11	bpa	–	40	15	2	35	22	<1	13
12	bpa	Hpic	40	15	2	49	32	<1	16
13^c^	bpa	.	40	15	2	18	6	–	12
14^c^	bpa	Hpic	40	15	2	35	21	–	14
15	bpa	–	60	15	2	39	25	<1	14
16	bpa	Hpic	60	15	2	51	36	<1	15

a *Experimental conditions: [Fe] = 1.5 × 10^−2^ M; [sub]/[Fe] = 50; H_2_O_2_(aq) 30%; solvent = water. FA, formic acid; DHA, dihydroxyacetone; GA, glycolic acid; HPA, hydroxypyruvic acid <1% in all reactions*.

b *[cocat]/[Fe] = 5*.

c *[L]_tot_/[Fe] = 5*.

Complex **3** showed a limited solubility in water, therefore it was tested as catalyst precursor only at 40°C or higher: it showed a moderate catalytic activity and a low selectivity in terms of DHA formation, the only other (and main) product formed being formic acid (see Table 4, entries 1-3). The addition of the potential cocatalyst Hpic caused a moderate increase of overall conversion but no significant change of selectivity (in Table 4 compare entries 4 and 3). In order to ascertain whether the catalytic results were limited by incomplete catalyst solubilization, some reactions were repeated using a 1:1 acetonitrile-water mixture, producing similar amounts of formic acid but lower amounts of DHA, with respect to the corresponding reactions performed in water.

Complex **4** proved to be soluble at r.t. in water. The first catalytic tests carried out at 25°C showed an appreciable extent of glycerol oxidation together with an encouraging DHA yield (*e.g.*, with 2 eq oxidant, 33 and 10%, respectively; see Table 4, entry 5). Reactions in the presence of **4** were usually carried out for only 15 min as longer reaction times determined a loss in DHA selectivity. Interestingly, the addition of 3 eq of the free ligand bpa caused a decrease of formic acid but no loss of DHA yield which were 5 and 11%, respectively (entry 6 in Table 4); when the latter reaction was repeated using an increased amount of H_2_O_2_ the yields of both products increased to 11% (FA) and 14% (DHA), respectively (in Table 4 entry 7).

The effect of the additives (5 eq) Hpic, H_2_pca, and Me-Hpca was then explored: by comparison of entries 8, 9, and 10 with entry 5 (Table 4) a net increase in the overall conversion with all cocatalysts tested can be noticed, with the highest selectivity in DHA obtained with the addition of Hpic (31% FA and 16% DHA). Curiously, the addition of H_2_pca and Me-Hpca caused an increase in yield of glycolic acid, whereas the use of Hpic did not.

Catalytic reactions performed in the presence of **4** and higher temperature (40°C) gave similar results (in Table 4, entries 11-13) to the corresponding reactions at r.t., thus confirming the roles of the cocatalyst (increase of yields of both products) and added ligand (partial suppression of formic acid formation). Notably, by combining the two additions, *i.e*., both Hpic and the added ligand, the two effects appear to cancel each other out (in Table 4, compare entry 14 con 11).

Finally, reactions catalyzed by **4** at 60°C (entries 15 and 16 in Table 4) gave yields and selectivities with minor variations with respect to those performed at lower temperatures.

From the results above reported, although all the iron complexes examined proved to be active catalysts for glycerol oxidation, only the bpa derivative **4** showed a fair selectivity toward the most valuable product DHA. Notably, such a product was only obtained in 10–16 % yield, however formation of formic acid as a single other product looked to be a favorable feature in terms of possible isolation of DHA from the final reaction mixture. For a comparison of the catalytic results obtained with complexes **1**-**4** see Supplementary Material, Tables 1, 2.

With the purpose of proving the feasibility of DHA isolation after completing the catalytic reaction, higher scale tests were carried out in the presence of catalyst **4**. In a typical test, a reaction was performed with experimental conditions similar to those of entry 5 in Table 4, but with 4-fold amounts of substrate, catalyst, and hydrogen peroxide: the last component was added to the reaction mixture in two portions (the second one after 15 min) in order to minimize overoxidation. After 30 min from the first oxidant addition, sodium sulfite was added in order to eliminate H_2_O_2_ residues, then after filtration the solvent was removed under vacuum. Isolation of DHA was carried out by means of column chromatography on silica gel using acetonitrile as the mobile phase. Finally, solvent removal of the appropriate fractions gave DHA as a pure colorless residue (see material and methods section).

### Role of the Cocatalyst in Iron-Promoted Oxidation: Spectroscopic and ESI-MS Studies

**Figure 5:**
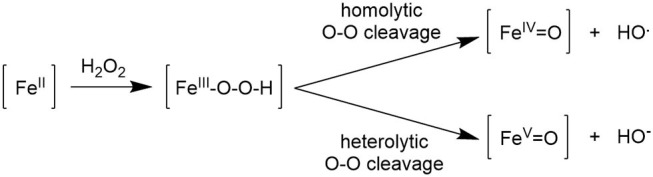
Proposed mechanism for the reaction of an iron(II) complex with hydrogen peroxide.

Several studies concerning the mechanism of iron-promoted oxidation using peroxides were published in the last two decades (Shi et al., 2008; Shejwalkar et al., 2011; de Visser et al., 2013; Hölzl et al., 2017; Song et al., 2017), which are aligned in proposing the key steps reported in Figure 5. Activation of the oxidant by formation of the intermediate Fe(III)OOH, itself a poor catalyst, is followed by the cleavage of the O-O bond, which can take place either in a homolytic or heterolytic fashion. The former path results in the formation of hydroxyl radicals, which brings about a radical Fenton-type reaction, whereas, the heterolytic path leads to an iron(V) oxo compound which is considered to be the catalytically active species responsible for metal-centered oxidation. The catalyst design aimed at tuning activity and selectivity by modifying the nature of coordinated ligands may be effective only when the poorly selective radical mechanism is suppressed in favor of the heterolytic path, which can in principle promote selective catalysis.

On the other hand, in spite of various investigations concerning the use of heterocyclic amino acid additives in iron-based oxidation (Shul'pin, 2002, 2013; Fernandes et al., 2011; Join et al., 2011; Kirillov and Shul'pin, 2013; Tanaka et al., 2014; Cozzi et al., 2018), the nature of their effect in such a reaction is still unclear. Also in the present study we observed that in some (although not in all) cases the use of the additives Hpic, H_2_pca, and Me-Hpca caused an increase of reaction yields. Thus, with the aim of gaining some information on the role of the co-catalyst in the series of reactions under investigation, we undertook a series of spectroscopic and MS studies concerning the evolution of complex **4**, *i.e.*, the most active and selective catalyst for glycerol oxidation, caused by the addition of the cocatalyst and, when applicable, the oxidant.

**Figure 6:**
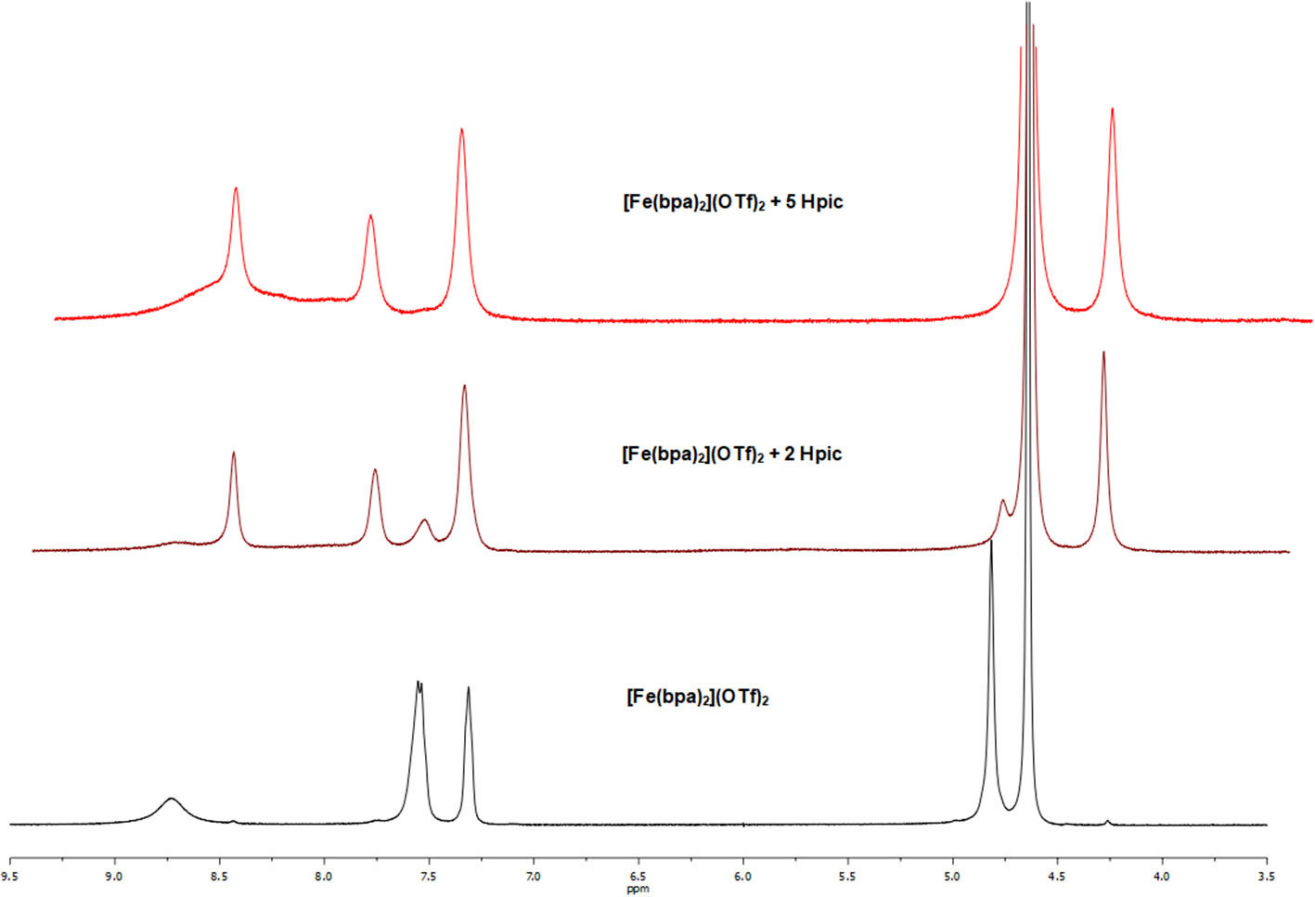
^1^H NMR spectra (D_2_O, 25°C) of [Fe(bpa)_2_](OTf)_2_ (**4**) with and without the addition of Hpic.

NMR studies were performed by recording the ^1^H NMR spectra of a solution of complex [Fe(bpa)_2_](OTf)_2_ (**4**) in D_2_O before and after the addition of Hpic. First of all, the addition of the substrate (1-phenylethanol or glycerol) caused no change in the resonances of **4**, suggesting that alcohol coordination did not take place. Then, when 2 eq of the additive were employed (either in the absence or presence of alcohol), the ^1^H NMR spectrum showed two sets of resonances: aside from the signals of complex **4** (δ 8.72, 7.55, 7.30, and 4.79) new resonances were observed at δ 8.44, 7.79, 7.36, and 4.24, all ascribable to a single compound according to COSY data, with a relative intensity of **4**: new species of 2:3. The experiment was repeated by using a higher amount (5 eq) of Hpic: the ^1^H NMR spectrum recorded immediately after amino acid addition showed only the resonances of the new complex, whereas the signals of complex **4** had disappeared (see Figure 6); moreover, the presence in the ^19^F NMR spectrum of a resonance at δ−78.9 indicated that, also in the newly formed species, triflate ion was not coordinated to iron. The addition of hydrogen peroxide to the solution of complex **4**, either in the absence or in the presence of added Hpic, gave rise in the ^1^H NMR spectrum to very large signals, due to the formation of paramagnetic species, of little use for identification of the iron derivatives formed.

**Figure 7:**
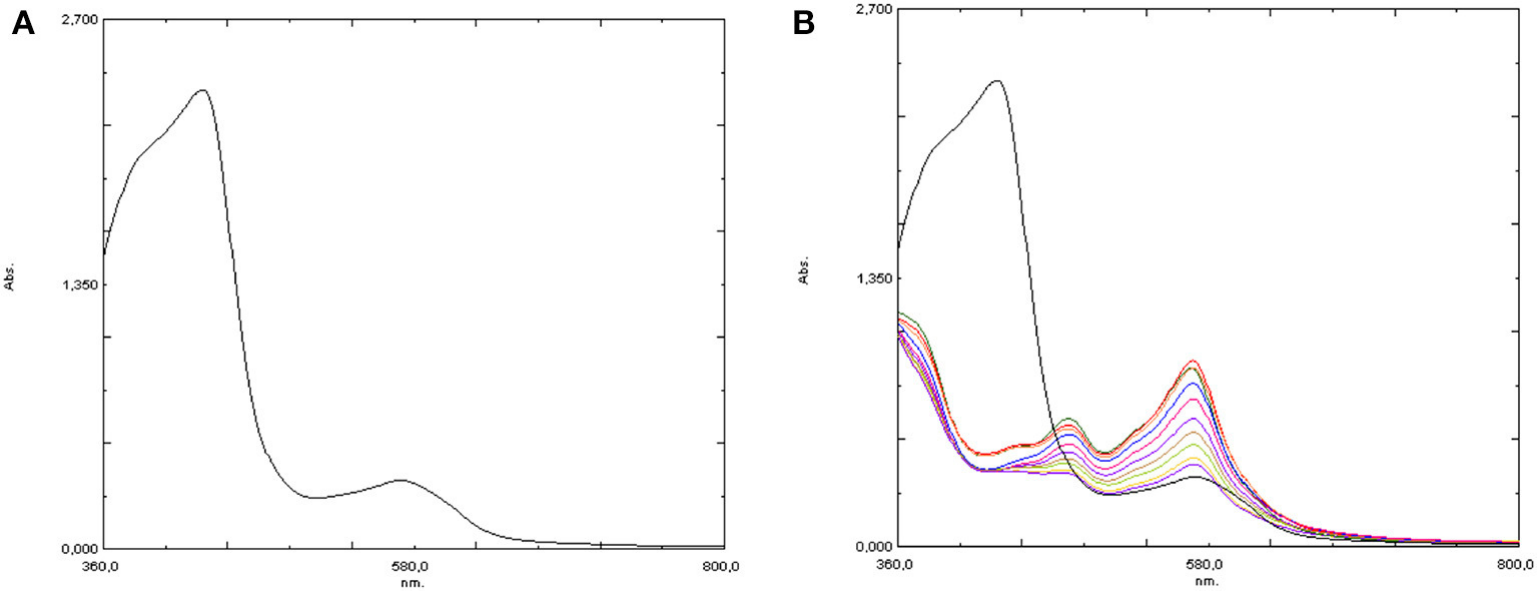
UV-visible spectra of [Fe(bpa)_2_](OTf)_2_ (**4**) in water **(A)** before and **(B)** after the addition of 10 eq H_2_O_2_.

On the other hand, UV-visible spectroscopy was potentially more suitable to follow the evolution of the iron complex after oxidant addition, either with or without the added Hpic. Therefore, the spectrum of **4** in water at 25°C was first acquired, showing a band at 430 nm (ε = 6,100 M^−1^cm^−1^) assignable to a metal-to-ligand charge transition (MLCT). Moreover, the presence of a second absorption of much lower intensity at 575 nm was also observed (see Figure 7A). The addition of the substrate (1-phenylethanol or glycerol) caused no spectral change, coherently with what was observed in the NMR studies. Then, the effect of the addition of 10 eq of hydrogen peroxide was followed by recording a sequence of spectra at intervals of 1 min: oxidant addition caused the immediate disappearance of the band at 430 nm, which was replaced by two absorptions at 481 and 570 nm, which in turn decreased rather rapidly with time (see Figure 7B). Notably, according to the literature (Kaizer et al., 2003; Mairata i Payeras et al., 2004; Seo et al., 2007; He et al., 2011) the band at 570 nm can be assigned to a Fe-OOH species, *i.e.*, the iron-hydroperoxide adduct which is the proposed intermediate for this class of reactions.

**Figure 8:**
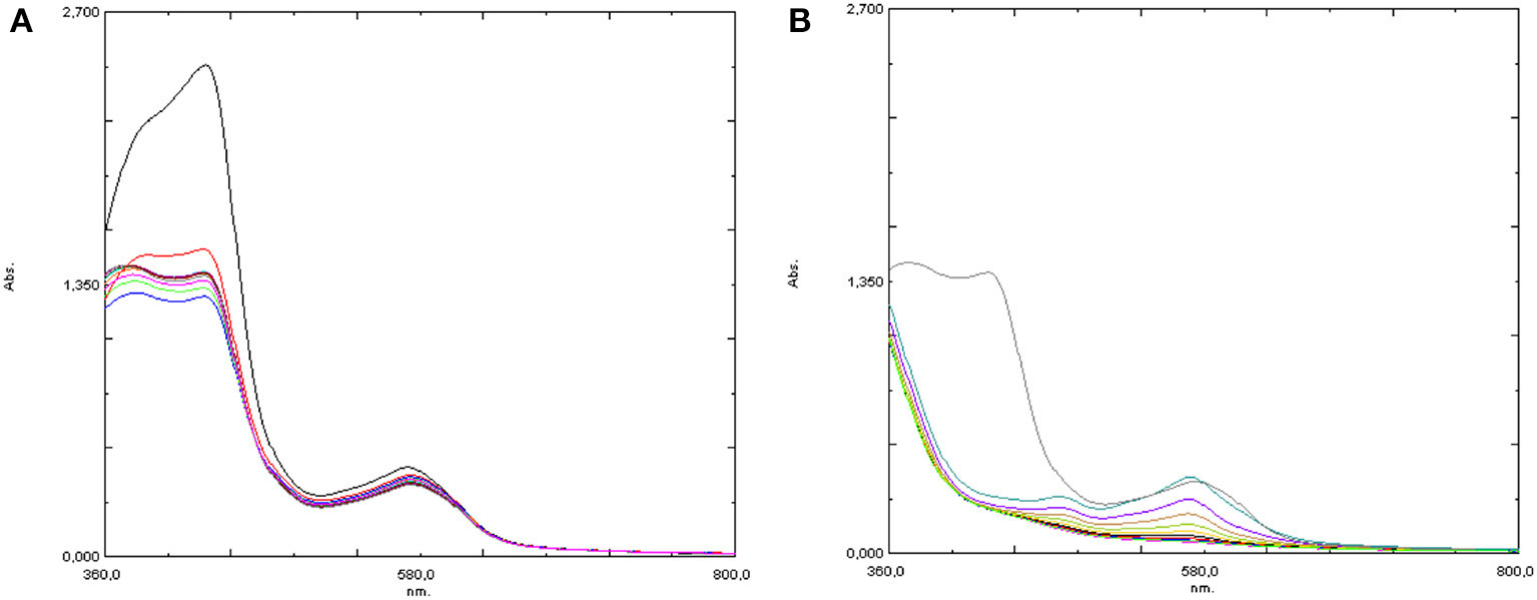
UV-visible spectra of [Fe(bpa)_2_](OTf)_2_ (**4**) + 2 eq Hpic **(A)** before and **(B)** after the addition of 10 eq H_2_O_2_.

In a second experiment, by treating the initial red-orange water solution of **4** with 2 eq of Hpic an immediate change of the color to orange was observed: the UV-visible spectrum showed a clear intensity decrease of the absorption at 430 nm, with no further change in the next 10 min (see Figure 8A). Then, hydrogen peroxide was added: the solution turned immediately violet and the evolution was followed by recording a series of spectra as in the analogous experiment in the absence of the additive, showing a formation of the same two absorptions at λ = 570 and 481 nm, which however in the presence of Hpic had lower intensity and decreased more rapidly than in its absence (Figure 8B).

**Figure 9:**
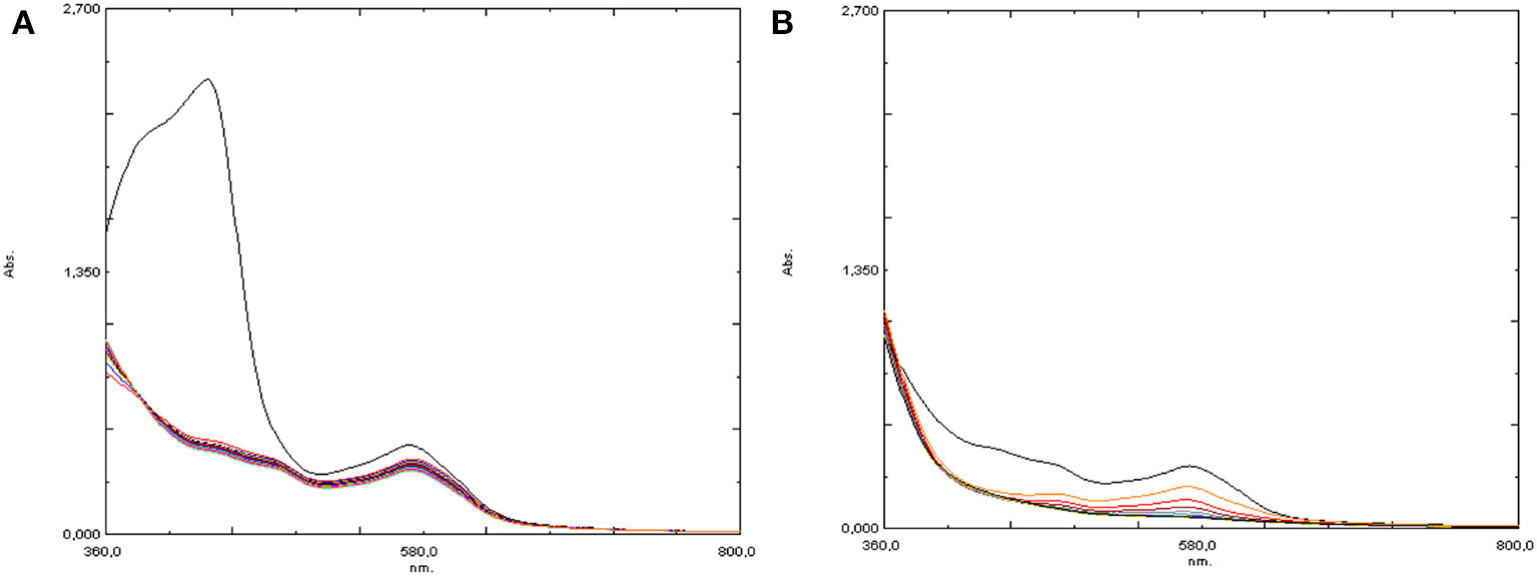
UV-visible spectra of [Fe(bpa)_2_](OTf)_2_ (**4**) + 5 eq Hpic **(A)** before and **(B)** after the addition of 10 eq H_2_O_2_.

A third experiment reproduced the second one but an increased amount of Hpic (5 eq) was employed: after amino acid addition, nearly complete disappearance of the absorption at 430 nm was observed (see Figure 9A); then, the addition of H_2_O_2_ caused the formation of the bands at 570 and 481 nm, although with low intensity, which rapidly decreased, disappearing within a few minutes (Figure 9B).

Following the same approach, we also studied, by ESI-MS, the evolution of complex **4** after the addition of 10 eq of H_2_O_2_ either with or without added Hpic. The ESI-MS spectrum of a solution of [Fe(bpa)_2_](OTf)_2_ (**4**) in water showed, besides signals related to free bpa ligand, a main signal at *m/z* 404 (Fe(bpa)(OTf)^+^), whereas the expected signal at *m/z* 603 ([Fe(bpa)_2_](OTf)^+^), reported elsewhere (Lenze et al., 2013a), was not detected, probably because a different ionization source was employed in those studies. The addition of H_2_O_2_ to the solution of **4** caused the immediate disappearance of the signal at *m/z* 404, whereas the presence of a weak iron-containing signal at *m/z* 509 suggested the formation of a new complex with oxidized bpa ([Fe(bpaox)_2_] + H)^+^ (bpaox = N-(2-Pyridinylcarbonyl)-2-pyridinecarboxamide), in agreement with the results of studies by Bauer and coworkers (Lenze et al., 2013a).

On the other hand, when the water solution of **4** was treated with 5 eq of Hpic the signals related to complex **4** disappeared, and in the resulting spectrum two new iron containing signals emerged (*m/z* 591 and *m/z* 639), which unfortunately we were unable to assign to a reasonable fragment. The addition of hydrogen peroxide caused an overall dispersion in the MS spectrum leading to a multitude of low intensity signals (see Supplementary Material).

Thus, NMR, UV-visible, and ESI-MS studies regarding the effect of Hpic addition to a water solution of **4** seem to provide coherent indications toward the formation of an adduct of complex **4** with the amino acid, which requires an excess (5 eq) of the latter to be completed. Notably, such findings are in contrast with the results of similar studies carried out in our laboratory concerning the effect of Hpic addition to a solution of [Fe(bpy)_3_](OTf)_2_ (**1**), as in such a case no spectral (NMR and UV-visible) change upon amino acid addition was observed, suggesting that no Hpic adduct with **1** was formed (Cozzi et al., 2018).

Besides the direct interaction between complex **4** and Hpic, another possible role of the additive might be the formation of peracids upon treatment with H_2_O_2_, as reported in the literature (Swern, 1953; Payne, 1961). However, our results as well as the reports by several other authors (Jain and Bhattacharyya, 2005; Kirillov and Shul'pin, 2013) provide no evidence of oxidation of the carboxylic group of Hpic in the presence of iron complexes.

On the other hand, the evolution of complex **4** after treatment with the oxidant, followed by UV-visible spectroscopy, appeared to be qualitatively similar either in the absence or presence of Hpic, although, when the latter was added, degradation of the intermediate formed was faster. With appropriate prudence due to the different concentrations employed in the spectroscopic experiments and the catalytic reactions, it is possible to suggest that the higher catalytic activity observed in the presence of the additive might be related to the faster evolution of the intermediate, *i.e.*, the iron-hydroperoxo species.

Unfortunately, all attempts to isolate and identify the adduct formed by the reaction of **4** with Hpic were unsuccessful, due to rather fast degradation of the corresponding solution when stored at a low temperature.

## Conclusions

In this paper we have described the highly sustainable oxidation of alcohols by hydrogen peroxide in water, catalyzed by iron complexes with nitrogen chelating ligands. The use of the novel ligand bpydeg, in comparison to unsubstituted bpy, enhanced the catalytic properties of the corresponding iron complexes for the oxidation of 1-phenylethanol in an aqueous medium. On the other hand, the oxidation of glycerol was studied by the use of four iron complexes with as many different nitrogen ligands, yielding in some cases appreciable amounts of the desired product DHA. Interestingly, the use of heterocyclic amino acids as cocatalysts in the latter reaction increased the reaction yields. The effect of such additives was explored by following the evolution of the iron complex [Fe(bpa)_2_](OTf)_2_ after the addition of Hpic by means of NMR and UV-visible spectroscopy as well as ESI-MS spectrometry: such studies indicated that the formation of an iron adduct with the amino acid took place, although identification of the latter could not be accomplished.

We believe that the present investigation provides a contribution toward the development of a more sustainable catalytic oxidation of alcohols, which makes use of the greenest possible oxidant (hydrogen peroxide) and solvent (water); moreover, our studies regarding the use of heterocyclic amino acids as cocatalysts might hopefully contribute to a better understanding of the largely debated effect of additives in catalytic oxidations.

## Data Availability Statement

All datasets generated for this study are included in the article/Supplementary Material.

## Author Contributions

DR carried out the ligand synthesis and the catalytic experiments. TG planned and supervised the ligand synthesis. EF coordinated the project and supervised the studies on iron complexes. CC performed the analysis and interpretation of the ESI-MS data. All authors contributed to the article and approved the submitted version.

## Conflict of Interest

The authors declare that the research was conducted in the absence of any commercial or financial relationships that could be construed as a potential conflict of interest.
