# Supplementary material for: Iron-Catalyzed Oxidation of 1-Phenylethanol and Glycerol With Hydrogen Peroxide in Water Medium: Effect of the Nitrogen Ligand on Catalytic Activity and Selectivity
Source: Front Chem. 2020 Oct 9;8:810. doi: 10.3389/fchem.2020.00810 (PMC7581906; doi:10.3389/fchem.2020.00810)
Supplement: Supplementary file 1 [file Table_1.DOCX]

Supporting Information for

**Iron-catalyzed oxidation of 1-phenylethanol and glycerol with hydrogen peroxide in water medium: effect of the nitrogen ligand on catalytic activity and selectivity**

**Dimitri Ros^a^, Teresa Gianferrara^a^, Corrado Crotti^b^ and Erica Farnetti*^a^**

^a^ Dipartimento di Scienze Chimiche e Farmaceutiche, Università di Trieste, Via L. Giorgieri 1, 34127 Trieste (Italy).

^b^ CNR – Istituto Struttura della Materia, Unità Operativa di Supporto di Trieste, S.R.14, Km163.5, 34149 Basovizza, Trieste (Italy).

* Corresponding author. Phone: +39-040-5583938; Fax: +39-040-5583903.

E-mail addresses: farnetti@units.it (E. Farnetti); gianfer@units.it (T. Gianferrara); crotti@ism.cnr.it (C. Crotti).

**^1^H NMR spectra of bpydeg and [Fe(bpydeg)_3_](OTf)_2_ (CD_3_CN, 25 °C)**

~~
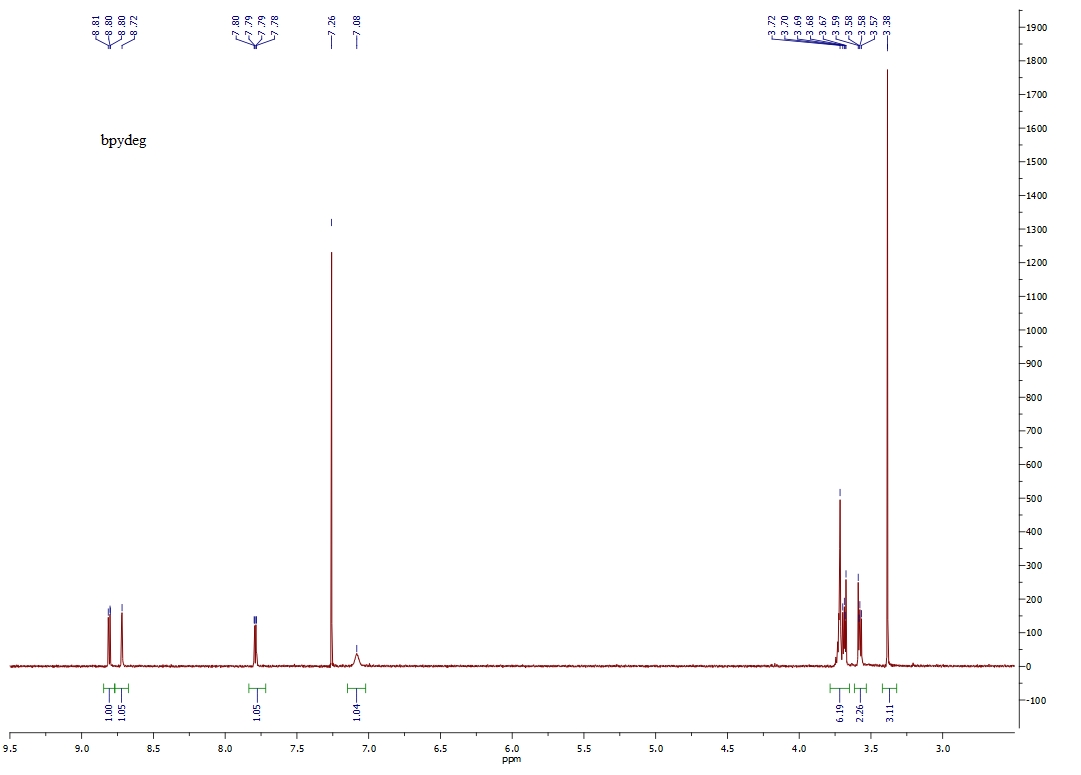
~~

~~
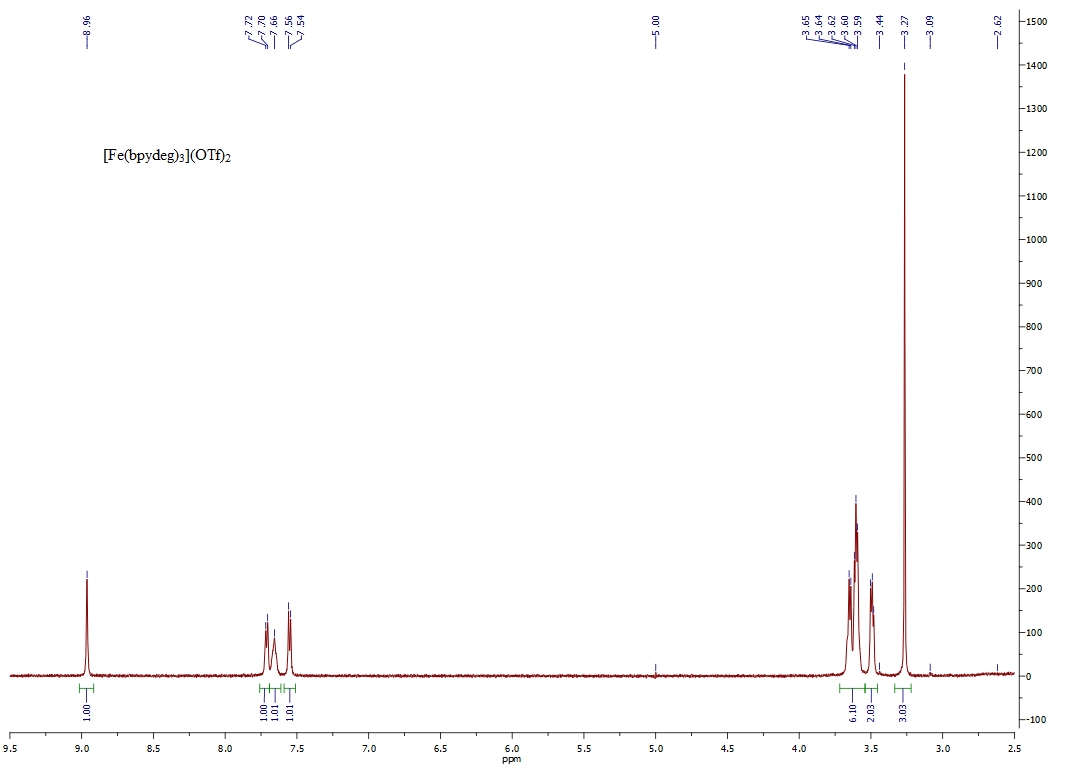
~~

**ESI-MS spectra: sample preparation and spectra acquisition**

**ESI-MS spectra of [Fe(bpa)_2_](OTf)_2_ + H_2_O_2_ in water**

A water solution containing either [Fe(bpa)_2_](OTf)_2_ ([Fe] = 2.0x10^-4^ M) was analyzed by MS.

1) ESI-MS spectrum of [Fe(bpa)_2_](OTf)_2_ (**4**) in water (solution A) in positive ion mode *before* H_2_O_2_ addition.

After addition of 10 eq of H_2_O_2_ a MS spectrum was immediately recorded, followed by a series of spectra at time intervals.

2) ESI-MS spectrum of solution A in positive ion mode immediately *after* H_2_O_2_ addition

3) ESI-MS spectrum of solution A in positive ion mode *after 10 min* since H_2_O_2_ addition

**ESI-MS spectra of [Fe(bpa)_2_](OTf)_2_ +Hpic + H_2_O_2_ in water**

In a second experiment, 5 eq of Hpic were added to a water solution containing either [Fe(bpa)_2_](OTf)_2_ ([Fe] = 2.0x10^-4^ M) and its MS spectrum was recorded afetr 15 min since the adding.

4) ESI-MS spectrum of [Fe(bpa)_2_](OTf)_2_ (**4**) in water in positive ion mode *after 15'* since Hpic addition (solution B).

Then, 10 eq of H_2_O_2_ were added to solution B and a series of MS spectra were recorded at time intervals.

5) ESI-MS spectrum of solution B in positive ion mode *after 2 min* since H_2_O_2_ addition

6) ESI-MS spectrum of solution B in positive ion mode *after 10 min* since H_2_O_2_ addition
