# Supplementary material for: Iron-Catalyzed Oxidation of 1-Phenylethanol and Glycerol With Hydrogen Peroxide in Water Medium: Effect of the Nitrogen Ligand on Catalytic Activity and Selectivity
Source: Front Chem. 2020 Oct 9;8:810. doi: 10.3389/fchem.2020.00810 (PMC7581906; doi:10.3389/fchem.2020.00810)
Supplement: Supplementary file 2 [file Table_2.DOCX]

Supplementary Material

# NMR spectra

~~
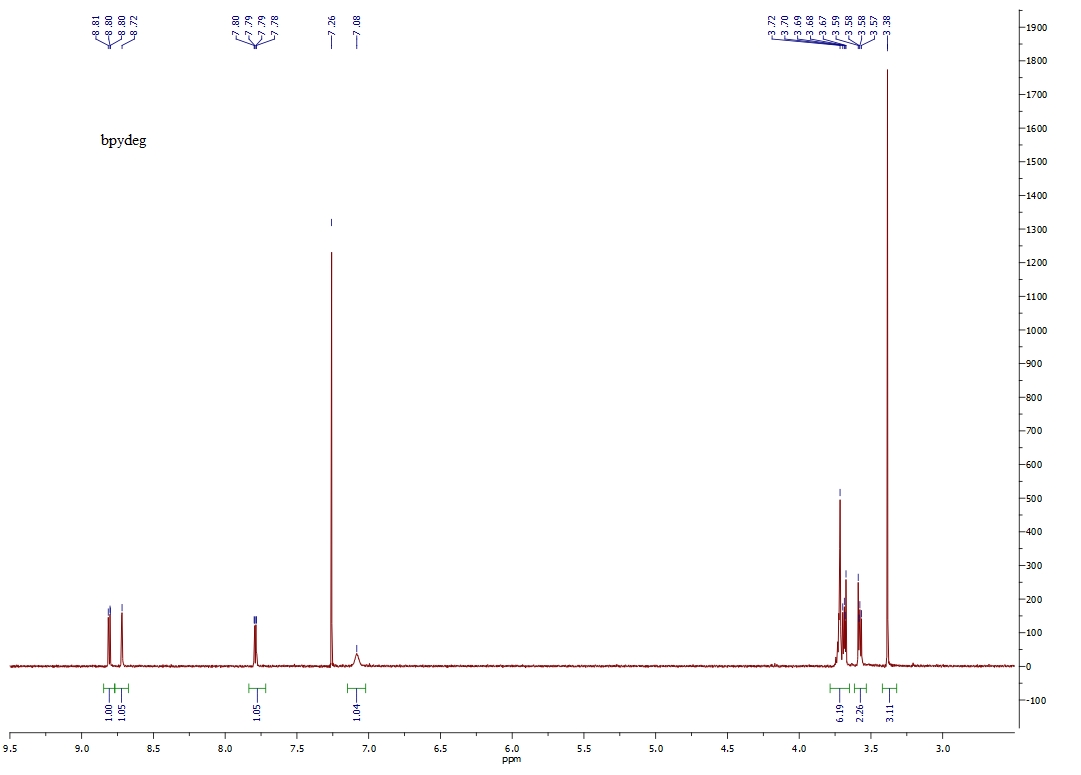
~~

**Supplementary Figure 1.** ^1^H NMR spectrum of bpydeg (CD_3_CN, 25 °C)

~~
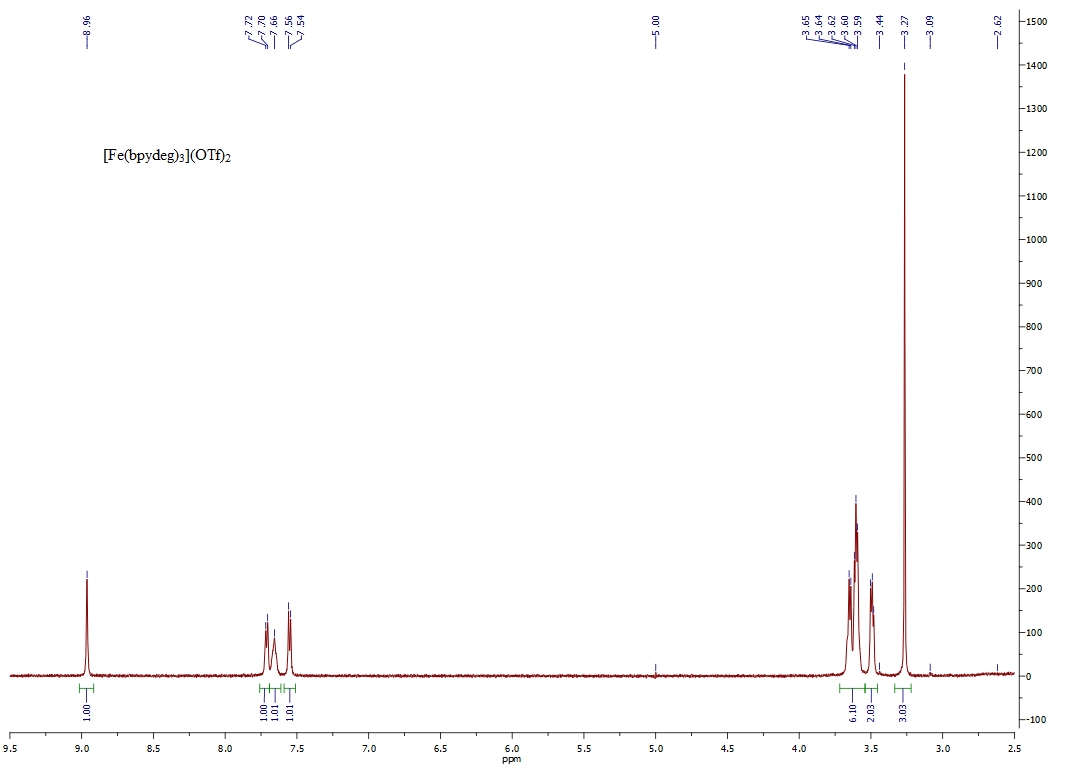
~~

**Supplementary Figure 2.** ^1^H NMR spectrum of [Fe(bpydeg)_3_](OTf)_2_ (**2**) (CD_3_CN, 25 °C)

# ESI-MS spectra: sample preparation and spectra acquisition

## ESI-MS spectra of [Fe(bpa)_2_](OTf)_2_ + H_2_O_2_ in water

A water solution containing [Fe(bpa)_2_](OTf)_2_ ([Fe] = 2.0x10^-4^ M) was analyzed by ESI-MS.

**Supplementary Figure 3.** ESI-MS spectrum of [Fe(bpa)_2_](OTf)_2_ (**4**) in water (solution A) in positive ion mode *before* H_2_O_2_ addition.

After addition of 10 eq of H_2_O_2_ an ESI-MS spectrum was immediately recorded, followed by a series of spectra at time intervals.

**Supplementary Figure 4.** ESI-MS spectrum of solution A in positive ion mode immediately *after* H_2_O_2_ addition

**Supplementary Figure 5.** ESI-MS spectrum of solution A in positive ion mode *10 min after* H_2_O_2_ addition

## ESI-MS spectra of [Fe(bpa)_2_](OTf)_2_ +Hpic + H_2_O_2_ in water

In a second experiment, 5 eq of Hpic were added to a water solution containing [Fe(bpa)_2_](OTf)_2_ ([Fe] = 2.0x10^-4^ M) and the ESI-MS spectrum was recorded 15 min after the addition.

**Supplementary Figure 6.** ESI-MS spectrum of [Fe(bpa)_2_](OTf)_2_ (**4**) in water in positive ion mode *15 min* *after* Hpic addition (solution B).

Then, 10 eq of H_2_O_2_ were added to solution B and a series of ESI-MS spectra were recorded at time intervals.

**Supplementary Figure 7.** ESI-MS spectrum of solution B in positive ion mode *2 min after* H_2_O_2_ addition.

**Supplementary Figure 8.** ESI-MS spectrum of solution B in positive ion mode *10 min after* H_2_O_2_ addition.

# Summary Tables of results for all complexes under optimized reaction conditions.

**Supplementary Table 1.** Oxidation of 1-phenylethanol.

| Catalyst | T (°C) | t (h) | TOF (h^-1^) | Conversion (%) | Selectivity (%) | Experimental conditions |
| --- | --- | --- | --- | --- | --- | --- |
| **1** | 100 | 0.5 | 134 | 67 | 100 | Table 1  entry 4 |
| **2** | 100 | 0.5 | 166 | 83 | 100 | Table 1  entry 7 |

**Supplementary Table 2.** Oxidation of glycerol.

| Catalyst | T (°C) | t (h) | TOF (h^-1^) | Conversion (%) | Selectivity DHA (%) | Experimental conditions |
| --- | --- | --- | --- | --- | --- | --- |
| **1** | 60 | 0.5 | 56 | 56 | 13 | Table 3  entry 9 |
| **2** | 60 | 0.5 | 54 | 54 | 11 | Table 3  entry 8 |
| **3** | 60 | 0.5 | 51 | 51 | 18 | Table 4  entry 4 |
| **4** | 25 | 0.25 | 32 | 16 | 69 | Table 4  entry 6 |
| **4** | 40 | 0.25 | 98 | 49 | 33 | Table 4  entry 12 |
